# Determination of Benzophenones in Water and Cosmetics Samples: A Comparison of Solid-Phase Extraction and Microextraction by Packed Sorbent Methods

**DOI:** 10.3390/molecules26226896

**Published:** 2021-11-16

**Authors:** Grażyna Wejnerowska, Izabela Narloch

**Affiliations:** Department of Food Analysis and Environmental Protection, Faculty of Chemical Technology and Engineering, Bydgoszcz University of Science and Technology, 3 Seminaryjna Street, 85-326 Bydgoszcz, Poland; izabela.narloch@pbs.edu.pl

**Keywords:** benzophenones, analysis of cosmetics, microextraction by packed sorbent, solid-phase extraction, water analysis

## Abstract

Benzophenones (BPs) are extensively used in a wide variety of cosmetic products and other materials (e.g., textiles or plastics) to avoid damaging effects of UV radiation. In the present work, we compared two extraction methods for the determination of BPs, namely, 2,4-dihydroxybenzophenone (BP-1), 2-hydroxy-4-methoxybenzophenone (BP-3) and 2,2-dihydroxy-4-methoxybenzophenone (BP-8), in water and cosmetics samples. The following extraction methods were used for the research: solid-phase extraction (SPE) and microextraction by packed sorbent (MEPS), whereas analysis was performed by gas chromatography with mass spectrometric detection. A comparison between the methods indicates that the MEPS technique(s) can be reliably used for analysis of BPs (sunscreen residue) in water samples and cosmetic samples with satisfactory results. This microextraction technique is cheap, easy, quick to implement, and consumes small amounts of solvents. On the other hand, the main advantage of the SPE method are low detection limits for the determination of BPs in water samples, i.e., from 0.034 to 0.067 µg L^−1^, while, for the MEPS method, LODs were at the level of 1.8–3.2 µg L^−1^. For both methods, the recoveries of BPs were 96–107% and 44–70% for water and cosmetics samples, respectively. The presented methods are suitable for use in cosmetics quality control and environmental pollution assessment.

## 1. Introduction

The ultraviolet (UV) filters, especially benzophenones (BPs), are most used in sunscreen products, cosmetics, lipsticks, hair sprays, hair dyes, shampoos, and other personal care products. Moreover, they can be found as additives in textiles, plastics, paints, car polishes, etc.

Benzophenone-3 (2-hydroxy-4-methoxybenzophenone (BP-3)), benzophenone-1 (2,4-dihydroxybenzophenone (BP-1)), and benzophenone-8 (2,2-dihydroxy-4-methoxybenzophenone (BP-8)) are very often used in sunscreens to protect human skin from ultraviolet radiation. Due to the high effectiveness of benzophenones and their appropriate properties, such as absorption or reflection of UV radiation in a wide range, they are chemically stable, e.g., they do not decompose in the cosmetic, under the influence of the sun or under the influence of other factors, do not evaporate after application, do not they cause staining of the skin, they do not smell, they are approved for use. Its recommended maximum content to 10% (*w*/*w*) in cosmetics has been formulated by appropriate legislation in many countries (Australia, Europe, China, and the Mercosur) [1,2,3,4].

These compounds can enter the aqueous environment directly or indirectly, for example, as a result of swimming and bathing in lakes and rivers, from showering, washing, and via wastewater treatment plants, by virtue of which they are ever-more present in environmental waters [4,5,6]. They are photostable, lipophilic, and potentially bioaccumulative compounds. The relatively high log octanol/water partition coefficient (log Kow > 3) value of BPs suggests its slow biodegradation and tendency to adsorb the suspended solids and sediments [4,7]. Nowadays, there is evidence to support the fact that BP-3 is absorbed through the skin and can bioaccumulate both in wildlife and humans [4]. Some of these compounds have been found in fish, urine, and breast milk [5,8,9,10]. In the last years, different toxicological studies conducted in vitro or in vivo in animals suggested that some of UV filters show significant estrogenic and/or antiandrogenic activity [4].

Among BPs, BP-3 is one of the most often detected in surface water from bathing areas [7]. When BP-3 is applied on the skin, it is partially absorbed by the human body and excreted as more polar metabolites, such as BP-1 and BP-8. They are also used as UV absorbers to protect goods against UV radiation. BP-1 is also the main metabolite of BP-3, identified in human urine. BP-8 is considered as a genotoxic compound [1,7]. Moreover, they are prone to evolve into halogenated by-products when mixed with chlorine ions [1,11,12]. The presence of BPs in the environment and their content in cosmetics should be monitored.

There are no official analytical methods for the determination of BPs in cosmetic products. In our previous literature report [13], we presented the methods used to measure BPs in water, urine, tissues, and cosmetics. To summarize, according to the literature data, the methods such as supercritical fluid extraction (SFE) in combination with capillary electrophoresis (CZE) and high-performance liquid chromatography (HPLC) [14] or polarographic method [15] are applied for the determination of BPs in cosmetic products. In the case of cosmetic samples, the method of gas chromatography coupled with MS is used very rarely [13]. Consequently, there is a great interest in the development of sensitive and selective analytical methods to ensure consumer health and the control of environmental pollution.

Gas chromatography and high-performance liquid chromatography in combination with mass spectrometry (GC-MS, HPLC-MS) is the most common method and it allows the accurate determination of UV filters in water samples [15]. Content of BPs in the environmental water samples is in trace amounts so that a preconcentration step must be carried out prior to their chromatography analysis.

For this purpose, the most common sample preparation techniques are used, such as liquid–liquid extraction (LLE) [16] and solid-phase extraction (SPE) [1,17,18], as well as microextraction techniques such as solid-phase microextraction (SPME) [19,20,21,22], single drop microextraction [23], dispersive liquid–liquid microextraction (DLLME) [6,24,25,26], stir bar sorptive extraction (SBSE) [27,28,29,30,31], microextraction by packed sorbent (MEPS) [32,33], and stir bar sorptive-dispersive microextraction (SBSDµE) [34]. The dispersive micro solid-phase extraction ((DI)µ-SPE) [35], fabric phase sorptive extraction (FPSE) [36], and pressurized liquid extraction (PLE) [37] are used also.

In the case of LLE and SPE, the main disadvantages are that it is time-intensive, uses large amounts of potentially toxic and expensive organic solvents, and requires high sample manipulation. Therefore nowadays, the so-called microextraction techniques play an important role in the sample preparation of environmental water for analysis, while microextraction methods such as SPME and SBSE use expensive, easy to damage materials and usually have carry over effects. However, they also have many advantages. SPME is fast, sensitive, solvent-free, and simple, whereas SBSE with thermal desorption is characterized by a very low limit of detection, while MEPS is a relatively new miniaturized SPE technique where the sorbent bed (1–4 mg) is integrated into the liquid handling syringe (100–250 µL). First, this technique is simple to operate, fast, inexpensive, precise, sensitive, environmentally friendly, and almost solventless [8]. Additionally, MEPS can be used for various types of matrices. Therefore, we decided to check the suitability of this method for the determination of BPs in cosmetics samples.

We applied MEPS and SPE techniques to compare both methods, especially in terms to determine BPs in complex matrices such as different cosmetics samples. To our knowledge this is the first paper reporting application of these methods prior to analysis by gas chromatography-mass spectrometry detection (GC-MS) to the separation and quantification of BPs in cosmetics products.

## 2. Results and Discussion

In the first stage of the studies, optimization of conditions of the chromatographic analysis (GC-MS) for the determination of BP-1, BP-3, and BP-8 (standard solution in methanol) was performed. The limits of detection of the analytes were determined, calibration curves were prepared, and precision of the chromatographic analysis was determined. All compounds showed good linearity (R^2^ > 0.984) by direct injection with a linear range of 2.5–600 µg L^−1^. The limits of detection (LODs), calculated as signal-to-noise ratio (S/N) of 3, ranged from 34 to 70 ng mL^−1^ for the MS(SCAN) detector and from 13 to 24 ng mL^−1^ for the MS(SIM) detector. The instrumental precision as relative standard deviations (RSD) was lower than 6.3% (at concentration of 100 µg L^−1^).

**Figure 1:**
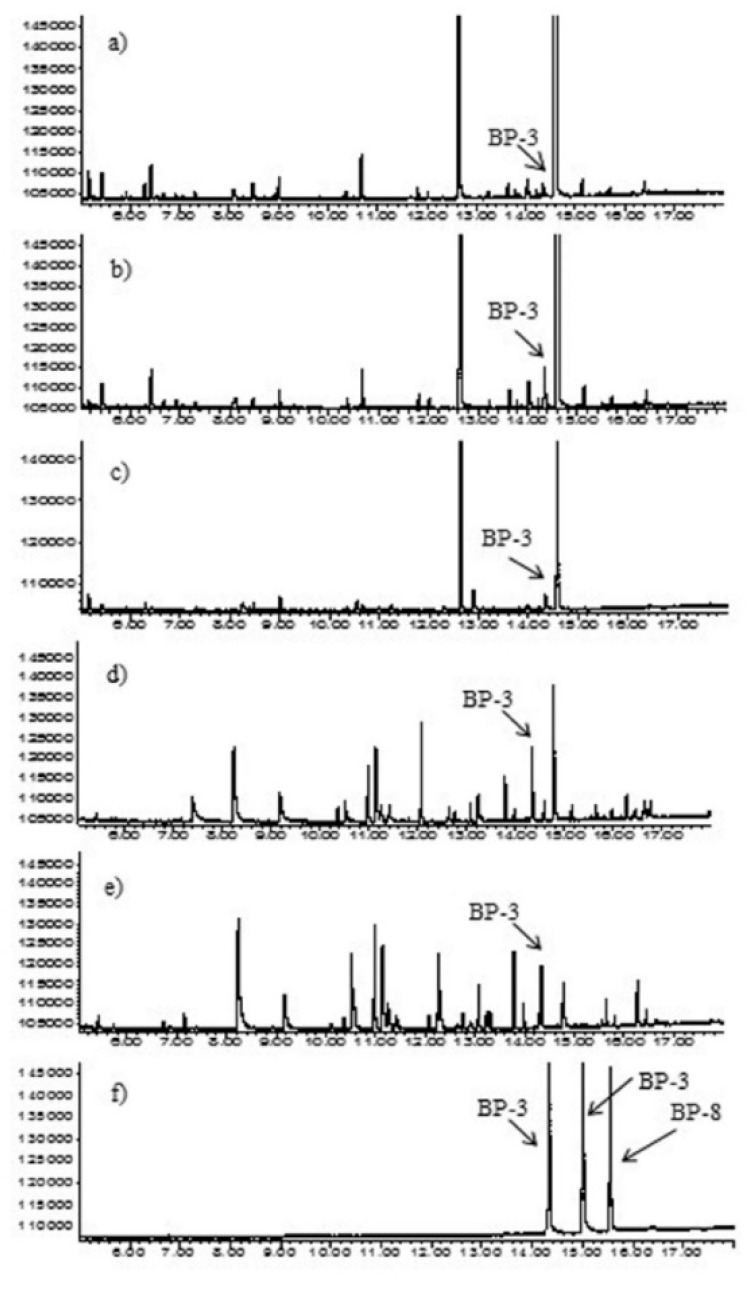
Chromatograms of (**a**) hair mask sample—SPE method; (**b**) hair mask sample with addition of BP-3 standard—SPE method; (**c**) hair mask sample—MEPS method; (**d**) shampoo sample–SPE method; (**e**) shampoo sample—MEPS method; (**f**) standard solution of BPs at concentration of 50 µg mL^−1^.

Satisfactory parameters of the chromatographic analysis allowed us to conduct research on the extraction methods. In the case of water samples, three benzophenones were tested: BP-1, BP-3, and BP-8. However, in the case of cosmetics samples, only one of the benzophenones—BP-3—was tested. The reason for this was that during inspection of cosmetics in local stores it turned out that only cosmetics containing BP-3 were available. However, the results of research indicate that BP-1 and BP-8 will behave during extraction similarly to BP-3. The selectivity of the method was assessed by the absence of interfering chromatographic peaks at the retention time of the target analytes (Figure 1).

### 2.1. Optimization of SPE Conditions

The determination of BPs in water samples using SPE (500 mg C18 cartridges) was performed according to the procedure presented by Giokas et al. [18], who obtained the recovery rate for BP-3 at the level of 95–97% for the natural water samples. Using this procedure, we obtained recovery rates for three BPs ranging from 101 to 107%. The accuracy, expressed as recovery percentage (%) of the SPE-GC-MS method, was calculated as the ratio of the found concentration to the expected concentration (concentration 5 µg L^−1^) after spiking a sample. The repeatability, expressed as relative standard deviation (%RSD) of peak areas, was evaluated by applying the proposed method in six replicates at two concentration levels (5.0 and 50.0 µg L^−1^) of standard solutions containing the target analytes. The intra- and inter-day precision values for all analytes in water samples were lower than 11.8 and 13.4%, respectively, highlighting the good reproducibility and repeatability of the method (Table 1). The accuracy and precision were satisfactory and therefore no modifications were introduced into the procedure.

To examine the enrichment factor (EF), the ratio of the final concentration of analytes in the solvent after extraction to the concentration of analytes in water solution subjected to the SPE process under optimum conditions was calculated. This value was also corrected by a degree of recovery. The use of large sample volumes (500 mL) results in a high enrichment factor (~1000), which has an impact on the possibility of determining BPs at low concentration levels, the values of which depend on the type of detection used. Parameters characterizing the SPE method are presented in Table 1. When using the MS detector, LODs were obtained at low concentration levels, ranging from 34 to 67 ng L^−1^. According to the literature data, 10 times lower LODs values can be obtained when using the MS-MS detector [20]. LODs values obtained for the SPE technique are comparable to other methods, i.e., SPME (0.15–8.2 ng L^−1^) [20,21], SBSE, and DLLME (2–11 ng L^−1^) [26,28], where BPs were derivatised and analyzed by GC -MS/MS.

### 2.2. Optimization of MEPS Conditions

BIN with C18 filling was used to investigate the possibility of using the MEPS technique for the determination of BPs in samples of water and cosmetics. As the investigations on the use of the C18 deposit in the SPE technique showed very high recoveries of ~100%, we decided to base on the parameters of this procedure. Basing on the procedure used for the SPE, ethyl acetate (EA) and dichloromethane (DCM) were used as the conditioning solvents (250 µL) and 100 µL of the EA/DCM mixture (1:1, *v*/*v*) for elution in the MEPS method. Using these parameters, the recovery was only 70–80% for BP-1 and BP-8 and 90% for BP-3.

**Figure 2:**
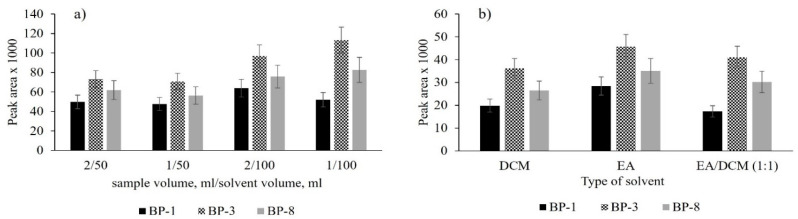
Optimization of MEPS method, sample spiked concentration 100 µg L^−^^1^, (**a**) effect of sample volume (1, 2 mL) and eluent volume (50, 100 µL) (EA/DCM, 1:1, *v*/*v*); (**b**) selection of eluent (sample volume 1 mL, eluent volume 100 µL).

For this reason, it was checked whether the sorption bed was overloaded (1 and 2 mL of sample) and whether the amount of eluent was sufficient to elute the analytes (50 and 100 µL) with the use of different eluents (DCM, EA, and EA/DCM mixture (1:1, *v*/*v*)). An effect of these variable parameters on the peak areas is presented in Figure 2.

For the graphic presentation of the effect of the sample volume subjected to extraction and the extractant volume (Figure 2a), the results were converted to equal values of these volumes.

The presented results indicate that no overloading of the bed was found with the larger sample volume (2 mL) introduced. On the other hand, the volume of the eluent used for desorption has the greatest influence on the extraction efficiency.

The greater volume of solvent (100 µL) makes the elution step more efficient. In addition, it was observed in subsequent studies that elution of analytes with two portions of solvent (2 × 50 µL) increased its efficiency by ~12% compared to one-stage elution (1 × 100 µL).

For the study on an effect of solvent type on the extraction yield, 100 µL each of DCM, EA and an EA/DCM mixture (1:1, *v*/*v*) were used. The results of studies are presented in Figure 2b. Studies have shown that the type of solvent has a significant effect on the desorption stage. The best desorption effects are obtained when EA is used. Its efficiency of desorption is approximately 20–40% higher than that of the other solvents used, therefore it was used in further studies.

At predetermined, optimal extraction conditions, the degrees of recovery and the precision of the method, expressed as relative standard deviation (%RSD, *n* = 6) of peak areas, were determined. The results of the studies are presented in Table 1. Satisfactory recovery rates of 90, 96, and 106% were obtained for BP-3, BP-1, and BP-8, respectively. The intra- and inter-day precision of the MEPS method is high for low concentrations, ranging from 11.8 to 18.2%, while for higher concentrations it is satisfactory and ranges from 4.0 to 11.2%.

It was found that the MEPS technique has one significant disadvantage, i.e., a very low enrichment factor of about 20. It results from a very small amount of the analysed sample, the possible increase of which will not cause a large increase in the value of the enrichment factor. Therefore, the MEPS technique can only be used to determine higher concentrations of analytes in test samples. Table 1 shows the LODs of the tested BPs. These values confirm the earlier assumptions, as the LODs were 1.8–3.2 µg L^−^^1^. Apart from the problem of the low enrichment factor, the MEPS technique has some very important advantages. These advantages are the small volume of solvent used and the small sample volume needed for the test. Additionally, MEPS is an easy, rapid (10 min), and not very labor-intensive process. The parameters of this method showing their advantages and disadvantages in comparison with other technics using GC-MS described in the literature are presented in Table 2.

When using SPE cartridges, the sorbent is discarded after use. In the MEPS method, the sorbent is used repeatedly. According to the manufacturer’s information and literature reports, depending on the sample matrix, the MEPS-BIN can extract up to 100 samples with stable efficiency. We have conducted sorbent stability studies by comparing the effectiveness of the used BIN to the effectiveness of a new, unused (after conditioning) bed. In the case of analysis of BPs in water samples after ~100 extractions, the efficiency of the bed decreased by ~10%. However, in the case of analysis of the cosmetics solution after ~70 extractions, the extraction efficiency decreased by ~20%, followed by the BIN exchange.

### 2.3. Application of SPE and MEPS Methods for the Quantitative Determination of BP-3 in Cosmetics Samples

The developed SPE and MEPS methods, as described above, can be successfully applied to the determination of BPs in water samples. However, we decided to check whether they would also be suitable for the determination of BPs in cosmetics samples.

An analysis of the composition of cosmetics available in local stores and containing UV agents was performed. It was found that BP-3 was commonly found in cosmetics from the group of benzophenones. In the first stage of the studies, a hair mask containing BP-3 and a shampoo without UV filters were used. A hair mask is a cosmetic with a much higher density compared to a shampoo. The first stage of study on the application of the developed methods for the analysis of BP-3 in cosmetics consisted in the selection of the cosmetic:water ratio. The following cosmetic:water proportions were applied: (*m*/*v*)—1:10,000 for SPE and 1:1500 for MEPS. The preparation of cosmetics solutions in water consisted in weighing them and then dissolving them by mixing the solution with a magnetic stirrer. Dense samples of cosmetics (hair mask) required long mixing of the solution (30 min) to dissolve them completely, while dissolving the shampoo was much faster (15 min). The samples prepared by this method were subjected to SPE and MEPS extraction according to the procedures developed for water samples. In order to check the selectivity of the method and the possibility of BP-3 detection, the extracts were analyzed by chromatographic method.

The previously used parameters of the chromatographic analysis turned out to be suitable also for the analysis of cosmetics samples. Figure 1 shows exemplary chromatograms of extracts obtained after the preparation of cosmetics samples using the SPE and MEPS methods. The identification of BP-3 was confirmed by the internal standard method (Figure 1b) and by analysis performed with the MS detector.

Recovery and relative standard deviation (RSD) are the most important parameters of the tested methods (SPE and MEPS), allowing their use for the quantitative determination of BP-3 in cosmetics samples. These parameters were determined by testing a shampoo without UV filters. Two samples of the shampoo were prepared to which BP-3 was added in amounts of 0.033 and 0.330% and then the samples were prepared according to the procedures described above. The results of tests are presented in Table 3.

The results of both intra- and inter-day precision expressed as relative standard deviation (%RSD) for SPE and MEPS methods ranged from 3.9 to 15.5%. Considering the low concentration of BP-3 in the tested solution and the type of matrix tested, i.e., a cosmetic, these values can be considered satisfactory.

The accuracy of the methods (expressed as recovery, R) was calculated as the ratio of the found concentration to the expected concentration after spiking a sample. It was examined at the two concentration levels; every level was examined in three separate experiments. The recovery depends on the BP-3 content of the shampoo sample. In both cases, a higher recovery was obtained for lower concentrations (0.033%), amounting to ~70%. In contrast, the higher BP-3 content in the shampoo resulted in a significant reduction in recovery to 44 and 58% for MEPS and SPE, respectively. The recoveries for BP-3 from the shampoo sample were lower than in the case of the water samples, which proves the influence of the matrix on their values. It can be observed that the recovery is much lower for MEPS compared to SPE. This is probably due to a small amount of the sorption bed, and thus to the higher sensitivity to ’matrix effects‘. The only solution is to prepare a water sample with a lower cosmetics content and to use a more sensitive detector, e.g., the MS-MS detector.

Due to the varied and ’rich‘ composition of cosmetics and relatively low levels of BP-3 recoveries (by SPE and MEPS methods), it was found that the most appropriate method for quantitative analysis of BPs in cosmetics would be the calibration method with standard addition (SA). The standard addition (SA) method is a powerful tool to minimize matrix effects and that enables precise and accurate determinations. It is also very important that the application of the SA method does not require the determination of recovery rate for each individual sample. However, it is laborious because it requires the preparation of a calibration curve for each sample. On the other hand, with respect to the MEPS method, in which the same sorbent is used many times, the phenomenon of the ‘wear’ of the bed will not have a major impact on the results.

A cosmetic (hair mask) containing information on the presence of BP-3 in its composition was used for the quantitative analysis. The quantitative analysis consisted in adding different amounts of BP-3 standard to the mask sample and analyzing these samples and the mask sample individually. The BP-3 content in the mask sample was calculated from the calculated value of the intersection of the calibration curve with the x axis.

The analyses were performed using two procedures (SPE and MEPS) for the same matrix in three repetitions. The linear correlation coefficients for the calibration curves for both methods were R^2^ > 0.99. Calibration curve equations and proportions of the prepared test samples were needed to calculate BP-3 content in the cosmetic (hair mask). The obtained mean results of studies were 0.059 and 0.065% for the SPE and MEPS methods, respectively. With the objective to demonstrate the equivalence in terms of precision and accuracy of the used methods, the Snedecor F-test and Student-t test were done. The results of the calculated parameters for both methods are presented in Table 4. No statistically significant differences were found between the precision and accuracy in the two methods.

**Figure 3:**
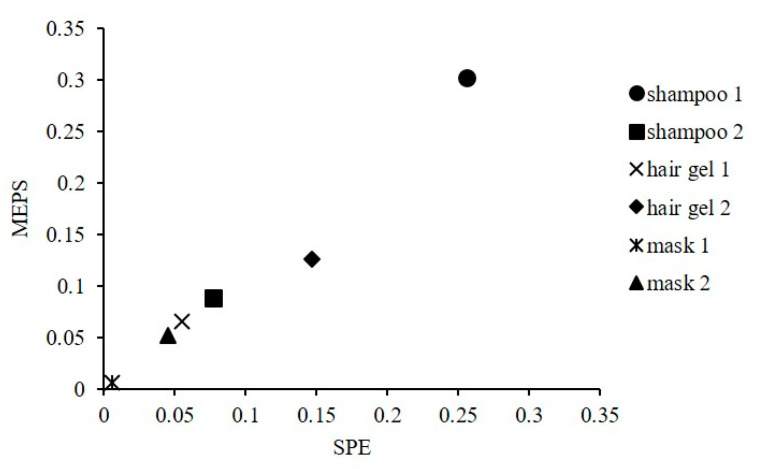
Comparison of BP-3 concentrations (%) in cosmetics samples determined by SPE and MEPS methods with GC-MS analysis.

The suitability of both methods for the determination of BP-3 in cosmetics samples with different composition was also confirmed. The tests were performed on the following cosmetics: two different shampoo samples and two different samples of a hair mask containing BP-3 and a hair gel to which BP-3 was added in two concentrations. Analyses of BP-3 content in these samples were performed by SPE and MEPS methods using the calibration method described above. The results of quantitative analysis are presented in the form of a graph (Figure 3).

A good correlation (R^2^ = 0.9676) was demonstrated between the results obtained by the two methods (SPE and MEPS). The results confirmed that both applied analytical methods are suitable for the quantitative determination of BPs in cosmetics.

In Table 5, the characteristics of the SPE and MEPS methods with the application of GC-MS and other analytical methods with the application of GC/MS-MS reported in the literature for the determination BPs in cosmetics are presented.

## 3. Materials and Methods

### 3.1. Materials and Reagents

BP-1, BP-3, and BP-8 were obtained from Sigma-Aldrich Co. (St. Louis, MO, USA). Their structures and relevant physico-chemical properties are given in Table 6. HCl (32%), which was used for pH adjustment, was from Chempur (Piekary Śląskie, Poland). Ethyl acetate (EA), dichloromethane (DCM), and methanol (MeOH) were purchased from Merck (Darmstadt, Germany). All compounds were analytical grade.

The BP-3 was determined in different cosmetic products: mask for hair and shampoo with BP-3; shampoo and hair gel without BPs. The cosmetic products were purchased from local shops.

### 3.2. Standard Solutions

Stock standard solutions (each compound~1.0 mg L^−^^1^) of BP-1, BP-3, and BP-8 were prepared in methanol and, additionally, a standard solution with BP-3 in methanol at a concentration of ~1.0 mg L^−^^1^ was used. These solutions were stored in the dark at 4 °C. From this standard solution, working solutions containing from 1.0 to 100.0 µg L^−^^1^ were prepared daily in water. The water solution was acidified (HCl) to pH 3.

The cosmetics products in amount of 0.1 g and 0.3 g (with accuracy to 0.0001 g) for SPE and MEPS methods, respectively, were spiked with standard solutions (BPs) of the appropriate concentrations and dissolved in 1000 mL water for the SPE method and in 500 mL water for the MEPS method. These solutions were mixed using the magnetic stirrer for 15–30 min and were prepared fresh every day.

### 3.3. SPE Procedure

The extraction of the analytes was performed using the C18 (1000 mg, 6 mL) cartridges obtained from Supelco (Bellefonte, PA, USA). The procedure was based as reported by Giokas et al. [30] and Lambropoulou et al. [17] with minor modifications.

The cartridge was conditioned with 5 mL EA and 5 mL DCM. Next, an aliquot of 500 mL of water or 100 mL of cosmetics solutions were pumped through the cartridge and air-dried under a vacuum. The analytes were eluted with 5 mL mixture of EA/DCM (1:1, *v*/*v*). The eluate was evaporated to dryness under a gentle stream of nitrogen at room temperature. The residue was redissolved in 0.5 mL EA and used in the GC analysis.

### 3.4. MEPS Procedure

Extraction was carried out by using a MEPS syringe (250 µL) packed with C18 (4 mg, mean particle size 45 µm, pore size 60 Å) sorbent from SGE (Trajan Scientific Australia Pty Ltd., Ringwood, Australia). Before being used for the first time, the packed sorbent was conditioned with 10 × 250 µL of EA, and then with 10 × 250 µL of DCM and 10 × 250 µL of EA/DCM (1:1, *v*/*v*).

The sorbent bed was conditioned by flushing 250 µL of EA/DCM (1:1, *v*/*v*) and 250 µL of ultrapure water before each extraction.

Next, 2000 µL of the sample was extracted by taking it from a vial and discarding to waste (eight cycles of 250 µL). Then, the sorbent was washed with ultrapure water (250 µL) and the cartridge was dried by pumping air through it (10 × 250 µL). The analytes were eluted with 100 µL of EA (2 × 50 µL). Finally, after elution the cartridge was washed three times with 250 µL of EA and three times with 250 µL of EA/DCM (1:1, *v*/*v*).

### 3.5. GC Analysis

Chromatographic analyses were performed using an Agilent 7890B (Agilent, Santa Clara, CA, USA), equipped with a split/splitless injector and multipurpose autosampler and an Agilent 5977B mass-selective detector.

The GC was fitted with a ZB-5-MS column (Zebron, Phenomenex Inc., Torrance, CA, USA), 30 m × 0.25 mm × 0.25 µm, containing (5% phenyl)-methylpolysiloxane.

The injector port was held at 270 °C and used in the splitless mode, and 2 µL injections were made. The temperature program used for the analysis was as follows: 100 °C, ramped at 10 °C/min to 260 °C and held for 4 min. Helium was used as the carrier gas at a flow rate of 1 mL min^−^^1^.

Full-scan mass spectra were recorded with *m*/*z* range 50–300 in electron-impact mode at 70 eV.

The transfer line and ion source temperatures were set at 280 and 230 °C, respectively. The scan rate was 2.9 scan/s, cathode delay time 5 min. The SCAN mode was used for optimization studies and identification of analytes. Identification was accomplished using the NIST Mass Spectral Database (NIST MS Search 2.3) and by comparing retention times with standards. The select ion monitoring (SIM) mode was used only for the determination of the limits of detection.

## 4. Conclusions

The aim of this work was to develop easy, environmentally friendly, and rapid analytical methods for the determination of BPs in water samples and consumer cosmetics products. The methods are based on gas chromatography analysis and sorption of BPs on the C18 bed.

The studies have shown that both methods used, i.e., solid-phase extraction (SPE) and microextraction packing solid extraction (MEPS), are fully useful for the determination of benzophenones in water and cosmetics samples. The microextraction technique MEPS is an alternative to SPE in terms of benzophenones in water and cosmetics samples.

Both methods are characterized by essential advantages, i.e., in the case of SPE a significantly lower limit of detection for analytes were achieved, while MEPS is a fast and simple method. Additionally, the use of organic solvents was drastically reduced. When determining BPs in cosmetics samples, it is very important to use the appropriate cosmetic:water proportions depending on the type of cosmetic and the expected BPs content in it. The applied calibration method with the standard addition is a guarantee of obtaining accurate results of quantitative analysis in cosmetics samples. Both of the methods are suitable for use in cosmetics quality control and environmental pollution assessment.

## Figures and Tables

**Table 1:** Characteristics of the SPE-GC-MS and MEPS-GC-MS methods for the determination of BPs in water samples.

Analytes	SPE							MEPS						
	Intra-Day (RSD, %) (*n* = 6)		Inter-Day. (RSD, %) (*n* = 6)		LOD ^b^, (µg/L)	Recovery ^a^, (%)	EF ^c^	Intra-Day (RSD, %) (*n* = 6)		Inter-Day (RSD, %) (*n* = 6)		LOD ^b^, (µg/L)	Recovery ^a^, (%)	EF ^d^
	5.0 (µg L^−1^)	50.0 (µg L^−1^)	5.0 (µg L^−1^)	50.0 (µg L^−1^)				5.0 (µg L^−1^)	50.0 (µg L^−1^)	5.0 (µg L^−1^)	50.0 (µg L^−1^)			
BP-1	9.0	7.7	10.8	8.0	0.034	101	1010	14.2	7.6	18.8	11.2	1.8	96	20
BP-3	8.2	11.2	8.6	11.0	0.050	105	1050	11.8	4.0	14.8	6.6	2.9	90	18
BP-8	11.8	11.0	10.9	13.4	0.067	107	1070	15.6	6.6	17.2	9.6	3.2	106	21

^a^ BPs at conc. of 5 µg L^−1^; ^b^ The determination limit (LOD) defined as three times the signal-to-noise ratio (*S/N* = 3); ^c^ Water volume 500 mL, eluent volume 0.5 mL; ^d^ Water volume 2 mL, eluent volume 0.1 mL; EF—enrichment factor.

**Table 2:** Comparison of proposed MEPS-GC-MS method to determine target analytes in water with other analytical methods reported in the literature.

Sample Preparation Technique	Matrix	LOD (ng L^−1^)	R (%)	RSD (%)	SAV ^a^ (mL)	SOV ^b^ (mL)	ET ^c^ (min)	EF	Reference
SPE-GC-MS/MS	water	0.3–1.0	67–73	1.8–3.0	100	6.1	-	700	[17]
SPE-GC-MS	water	3	95–97	5	500	20	60	50,000	[18]
MEPS-GC-MS	water	44.0–53.0	95–109	4–8	0.8	2	-	16	[32]
(DI)SPME-GC-MS/MS	water	0.15–3.0	80–115	6–13	10	-	30	-	[21]
(HS)SPME-GC-MS	water	9.0	-	<20%	40	-	125	-	[22]
(DI)SPME-GC-MS/MS	water	0.3–8.2	80–103	8.4–11	10	-	30	-	[20]
SBSE-LD-GC-MS	water, wastewater	2.0	28	1.3	100	0.2	510	140	[31]
SBSE-TD-GC-MS	water, wastewater	11.0	63	12–15	20	-	180	-	[28]
(DI)µ-SPE-GC-MS	water	0.5–2.0	85–96	4–9	10	-	10	-	[35]
SBSDµE-GC-MS	water	148	80–116	<12	25	-	50	-	[34]
FPSE-GC-MS/MS	water	4.5	88–110	9.2–12.0	30	20	3	-	[36]
MEPS-GC-MS	water	1.8–3.2	90–106	4.0–16	2	2	10	20	proposed method

^a^ SAV—sample volume; ^b^ SOV—solvent capacity; ^c^ ET—extraction time.

**Table 3:** Precision and accuracy of the SPE and MEPS methods obtained in determination of BP-3 in cosmetics samples.

Analytes	SPE						MEPS					
	Intra-Day (RSD, %) (*n* = 6)		Inter-Day (RSD, %) (*n* = 6)		Recovery (%)		Intra-Day (RSD, %) (*n* = 6)		Inter-Day (RSD, %) (*n* = 6)		Recovery (%)	
BP-3	11.5 ^a^	12.4 ^b^	13.8 ^a^	14.0 ^b^	69.5 ^a^	58.2 ^b^	3.9 ^a^	14.4 ^b^	6.6 ^a^	15.5 ^b^	69.7 ^a^	44.0 ^b^

^a^ 0.033% (BP-3 in shampoo); ^b^ 0.330% (BP-3 in shampoo).

**Table 4:** Statistical comparison between the two techniques by Snedecor *F*-test and Student-*t* test; determination of BP-3 content in hair mask sample.

Analyte	SPE Mean ± *s*_1_ (%)	MEPS Mean ± *s*_2_ (%)	*F* Ratio (*F*_cr_)	*t*-Values (*t*_cr_)
BP-3	0.059 ± 0.006	0.065 ± 0.004	2.25 (19.00)	1.440 (2.78)

*n*_1_ = *n*_2_ = 3; *v* = 4; For *α* = 0.05 critical *F* value = 19.0 and critical *t* value = 2.776.

**Table 5:** Comparison of proposed MEPS-GC-MS and SPE-GC-MS methods to determine target analytes in cosmetic samples with other analytical methods reported in the literature.

Sample Preparation Technique	LOD (%)	R (%)	RSD (%)	SAV ^a^ (mL)	SOV ^b^ (mL)	ET ^c^ (min)	Reference
GC-MS/MS	0.0018–0.27	101–105	0.69–1.13	0.1 g	0.7	40	[38]
PLE-GC-MS/MS	0.01–0.046	51.9–87.6	6.4–8.8	0.1 g	10	10	[37]
SPE-GC-MS	0.0003	58–70	12	0.1 g	15.5	60	proposed method
MEPS-GC-MS	0.001	44–70	14	0.3 g	2	15–30	proposed method

^a^ SAV—sample volume; ^b^ SOV—solvent capacity; ^c^ ET—extraction time.

**Table 6:** Characteristics of the UV filters studied.

Analyte	Molecular Formula	CAS Number	Structure	Log K_ow_	pKa
2,4-dihydoxybenophenone (BP-1)	C_13_H_10_O_3_	131–56-6	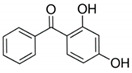	2.96	7.1
2-hydroxy-4-methoxybenophenone (BP-3)	C_14_H_12_O_3_	131-57-7	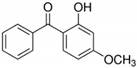	3.79	7.56
2,2′-dihydroxy-4-methoxybenzophenone (BP-8)	C_14_H_12_O_4_	131-53-3	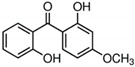	3.82	6.78

from: https://pubchem.ncbi.nlm.nih.gov (accessed on 30 October 2021).

## Data Availability

Not Applicable.

## Sample Availability

Samples of the compounds are not available from the authors.
